# Impact of WHO guidelines on trends in HIV testing and ART initiation among children living with HIV in Zambia

**DOI:** 10.1186/s12981-020-00277-0

**Published:** 2020-05-14

**Authors:** Tendai Munthali, Charles Michelo, Paul Mee, Crispin Moyo, Andrew Kashoka, Liswaniso Liswaniso, Innocent Chiboma, Jim Todd

**Affiliations:** 1 School of Public Health, University of Zambia, Lusaka, Zambia; 2 Ministry of Health, Lusaka, Zambia; 3 Department of Infectious Disease Epidemiology, Faculty of Epidemiology and Public Health, London School of Hygiene and Tropical Medicine, London, UK; 4 Department of Population Health, London School of Hygiene and Tropical Medicine, London, UK; 5 Equip Health, Lusaka, Zambia

**Keywords:** Childhood HIV, HIV testing, ART initiation, HIV treatment, HIV trends in Zambia

## Abstract

**Background:**

About 13 years since the introduction of antiretroviral therapy (ART) for children living with HIV (CLHIV) in Zambia, HIV/AIDS testing and treatment guidelines for children have evolved over the years with limited documentation of long-term trends in the numbers testing HIV positive and initiating ART. We examined trends in HIV testing and ART initiation in Zambia.

**Methods:**

We conducted a retrospective cohort study using routinely collected patient level data from 496 health facilities across Zambia. We used Poisson regression to derive incident rate ratios and 95% confidence intervals (95% CI) for background characteristics and used a Cuzick non-parametric test for trends to test the 13-year trends. Median time from testing to ART initiation in days and incidence rates were derived using life tables in survival analysis. We used multi-level random effects Poisson regression model to determine variations in time from HIV testing to ART initiation by facility.

**Results:**

Overall, the cumulative proportion of the children who tested positive and initiated antiretroviral therapy (ART for HIV) from 2004 to 2017 was 69% (n = 99 592). During the period under review proportions of ART initiation increased from 52% in 2004–2006 to 97% in 2016–2017 (P < 0.001) and time from testing to ART initiation reduced from a median of 17 days IQR (1–161) in 2004 to one day IQR (1–14), P < 0.001 in 2016–2017. CLHIV were 15 times more likely to be initiated on ART in 2016-17 compared to period 2004-6 (IRR = 15.2, 95% CI 14.7–15.7). Time to ART initiation increased with age and was higher in rural health facilities compared to urban facilities. About 11% of the variability in time to ART initiation in children could be attributed to differences between facilities.

**Conclusions:**

The substantial increase in ART initiation and reduction in time to ART initiation among CLHIV identified in this study, reflects improvements in the paediatric HIV programme in Zambia in relation to health care delivery and adherence to national testing and treatment guidelines that were adapted from WHO guidelines. However, age-related differentials in rates of ART initiation suggests that urgent interventions are needed to sustain and further improve programme performance.

## Background

Globally, there are about 37.9 million people living with HIV with about 24.5 million receiving antiretroviral therapy (ART) [1]. Among these, approximately 1.7 million are children under the age of 15 years, with only 54% of them receiving ART [1]. In Zambia there are 62, 000 children living with HIV (CLHIV) with 79% on ART and 3000 of them die from AIDS related illnesses annually [1, 2]. ART was first made available free of charge in Zambia in 2005 [3, 4] and since then treatment of children on ART has been based on the World Health Organization (WHO) treatment guidelines that are adapted for use in country. In 2006 WHO recommended initiating children on ART based on clinical staging (3/4) or CD4 + T cell counts (< 250 cells/μl/CD4% < 25%) [5]. The guidelines were then revised in 2008 recommending ART for all children less than 12 months regardless of CD4 count [6]. By 2010, WHO further revised the guidelines recommending immediate initiation on ART for all infants and children less than 2 years regardless of immunologic or clinical thresholds and for all children aged 2–5 years with clinical stages 3/4 or CD4 < 750 cells/μl or 25% [6]. In 2013, the WHO guidelines were revised to recommend immediate treatment for all children < 5 years old [7]. The current guidelines (2016) now recommend immediate initiation on ART for all HIV infected children regardless of age under the test and treat guidelines [8].

These changes in the guidelines have resulted in improvements in HIV testing and ART initiation among children [9–12]. However children still fall through the gaps and present for testing and ART initiation at older ages and in late HIV stages [11]. A study of 565 children initiating ART from 2004 to 2008 at one urban and two rural clinics in Zambia showed that about 50% of children started treatment between the ages 5 to 10 years [12]. Similar trends were seen in a study in Tanzania from 2004 to 2011 among children in 26 clinics where children were initiated on ART at a median age of 5.2 years [13].

In 2018, 13 years later after the introduction of ART in Zambia there are improved HIV testing services and better ART availability for CLHIV [14–16]. Studies conducted in sub-populations of children in Zambia have shown changes in the baseline characteristics of children testing HIV positive and initiating ART [14, 15]. However, little is known about long-term and country-wide characteristics of children diagnosed with HIV and initiating ART in Zambia. This paper uses national routine patient data to describe the baseline characteristics of children in Zambia from HIV testing to ART initiation and how these characteristics have changed over a 13-year period.

## Methods

### Study population

This study was a retrospective cohort study of children with records in the Zambian SmartCare data system. SmartCare is an electronic patient monitoring system which is used in many health facilities across all districts in Zambia [17–19]. Each patient is given a unique identification number at the first contact with the clinic and then subsequent records are entered into the database at each visit to health facility. The patient’s records are entered in SmartCare either electronically during the visit or after the patient has been attended to by entering the data from a paper-based patient file. SmartCare data is collected for routine patient monitoring but is prone to missing data in both the electronic database and in paper-based format. A total of 99,592, children less than 15 years of age at the time of HIV diagnosis with records in SmartCare regardless of missing data during the period under review were included in the analysis.

### Clinical procedures

In the Zambian health care system, all children with a positive serological test for HIV are eligible for enrolment into care. At the initial evaluation visit, a record is taken of medical history, physical examination, anthropometric measurements, socio demographic information and determination of WHO disease stage. All CLHIV should receive HIV care according to national testing and treatment guidelines which includes, the measurement of CD4 + T-cell counts or percentages, haemoglobin levels (HB), renal and liver function tests and HIV viral loads prior to starting ART. Treatment eligibility is determined based on the WHO and/or national treatment guidelines in effect at the time of HIV testing. Eligible CLHIV are treated with a first-line regimen and asked to return for clinical evaluation at week 2, week 4 and then every 3 months for stable children [20, 21].

### Data extraction and analysis

The data for this analysis were extracted from SmartCare and saved in Excel (Microsoft Corp, Seattle) format for cleaning. The data was then exported to Stata version 13 (Stata Corp, College Station, Texas) for analysis. Two date variables were used from the dataset, the date of the first HIV positive test result (HIV diagnosis) and the date initiated on ART. These dates were used to derive the year of HIV test, the year of ART initiation, and the time between HIV diagnosis and ART initiation. The time variables were grouped into the following year bands: 2004–2006, 2007–2009, 2010–2012, 2013–2015 and 2016–2017, which roughly correspond to the years when Zambia implemented the WHO guidelines for ART in children. The ages of the children were categorised into four categories: those less than 18 months, 18–59 months, 5–9 years and 10–15 years. In addition health facilities were categorised into Hospitals, health centres, hospices and health posts as classified by the Zambia Health facility listing [18].

### Statistical methods

Duplicate results were removed in Stata using the child’s ID, date of birth and relevant variables such as laboratory results, pharmacy visit and HIV test dates. For continuous data, a test for normality was performed and data that were not normally distributed were described using the median and inter quartile ranges (IQR), while normally distributed data were described using means and standard deviations. The two baseline characteristics: tested for HIV and initiated on ART were used as dependent variables. Frequencies and percentages were used for categorical data and the Cuzick non-parametric test for trends (which is a Wilcoxon-type test for ordinal data) was used to show the changes in proportions testing HIV positive across time for the different levels of the explanatory variables. Models for trend testing were selected based on type of independent variable. All variables that were tested for trends were ordinal and had two or more categories.

We declared the data as survival data using the time from HIV testing to initiation on ART using parametric survival analysis to estimate the rate of ART initiation. Dates when the child was enrolled into HIV care, the date of death for those that died or whether they were put on ART or not were used to censor the data in survival analysis. Incident rate ratios were derived using Poisson regression and a Wald test was used to assess significance. Mixed multi-level random effects regression modelling was used to show variability of time from HIV testing to ART initiation among the children attributable to type of facility used at ART initiation. Wald tests were used to compare goodness of fit for crude and adjusted models.

Cumulative aggregate numbers of children initiating ART from the UNICEF’s Stocktaking reports and the UNAIDS country reports published during the period under review were also compiled and compared to SmartCare numbers to validate trends in our analysis.

## Results

### Participants

**Fig. 1:**
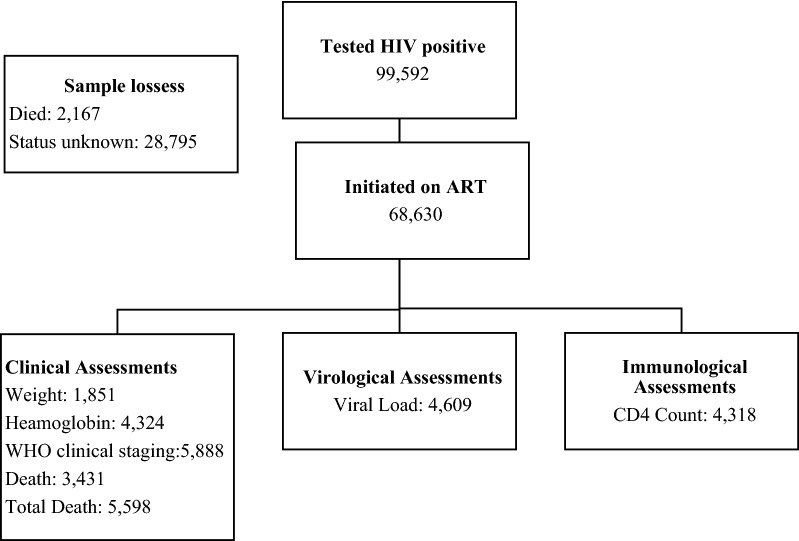
Flow diagram showing available records of children from testing to ART initiation from 2004 to 2017 December aged 15 years and below in Zambia

A total of 99,592 children were recorded as having a positive HIV test result from 496 health facilities across Zambia during the period under review. Of these children 52% (52,109) were female and 48% (47,483) were males. In this analysis a total sampling of all children in 496 health facilities and 71 districts from all ten provinces of the country with relevant records in SmartCare was conducted. Out of the 99,592 children recorded as having an HIV positive diagnosis between 2004 and 2017, 69% (68,630) had records showing they started antiretroviral therapy (ART). Overall mortality for the period under review was 6% (5817/99,592). Mortality in children who tested positive, was 7% before ART initiation (2167/31,097) and 5% (3431/68,630) in children after initiating ART. In addition, a total of 31% (31,097) of children that tested HIV positive had no record in SmartCare of ART initiation. For the children with records of ART initiation, after ART initiation, additional data on weight, height and clinical data are not well recorded or were missing in most cases, see Fig. 1.

The median age for HIV testing was 2 years with an interquartile range of (IQR) 1 to 4 years. Most of the records were from Lusaka (25%), Southern (17%), Copperbelt (21%) and Eastern provinces (10%). Western, Northern, Muchinga, Central, North-western and Luapula provinces each contributed less than 10% of the records, see Table 1.

**Table 1:** Baseline characteristics of children diagnosed with HIV from 2004 to 2017 in Zambia

Characteristic	Population	
	N	%
Sex		
Female	52,209	52
Male	47,483	48
Age of the children		
Age at HIV test Median (IQR)	2 (1–4)	
Province		
Lusaka	25,343	25
Copperbelt	20,701	21
Southern	16,498	17
Eastern	10,312	10
Central	7097	7
North-western	3664	4
Northern	3160	3
Luapula	3458	3
Western	7081	7
Muchinga	2277	2
Total	99,592	100

### HIV testing over time

The percentage of children testing HIV positive in the age groups from birth to 17 months and from 18 months to 4 years was highest in the period between 2007 and 2009 at 30% but declined steadily over the years. In contrast among the 10 to 15 age group, HIV testing was lowest in between 2007–2009 at 17% and increased over the years to 26% by the end of 2017. Median age at testing has remained high over the years with the lowest age at testing being 3 years IQR (1–8) in 2007–2009, see Table 2. Of those who tested HIV positive, most children (55%) were tested in hospitals in 2004–2006 however, this trend reduced to 37% between 2016 and 2017 with increasing testing in the other facilities (p-value < 0.001).

**Table 2:** Baseline characteristics of children at HIV positive diagnosis in Zambia from 2004 to 2017

Characteristic	2004/2006 n = 12,087	2007/2009 n = 30,848	2010/2012 n = 2917	2013/2015 n = 2028	2016/2017 n = 7212	P-value*
Age n (%)						< 0.001
Birth–17 months	1860 (16)	8948 (30)	8140 (28)	4499 (23)	1546 (22)	
18 months–4 years	3391 (29)	8936 (30)	7976 (28)	5431 (28)	1872 (27)	
5–9 years	3740 (32)	7074 (23)	6989 (24)	5183 (26)	1798 (26)	
10–15 years	2788 (24)	5227 (17)	5483 (19)	4535 (23)	1823 (26)	
Median age (IQR)	6 (2–10)	3 (1–8)	4 (1–9)	5 (2–10)	5 (2–11)	
Sex n (%)						< 0.001
Male	5868 (49)	15,018 (49)	13,740 (47)	9446 (47)	3411 (47)	
Female	6219 (51)	15,830 (51)	15,457 (53)	10,802 (53)	3801 (53)	
Place of testing n (%)						< 0.001
Hospital	6682 (55)	16,990 (55)	13,383 (46)	7759 (38)	2658 (37)	
Health Centre	5393 (45)	13721 (44)	15477 (53)	12,149 (60)	4217 (58)	
District type n (%)						< 0.001
Rural	811 (7)	3 791 (12)	4 538 (16)	3 395 (17)	836 (12)	
Urban	11,276 (93)	27,057 (88)	24,659 (83)	16,852 (83)	6376 (88)	
ART initiation status n (%)						< 0.001
Initiated on ART	6217 (52%)	16,940 (55%)	19,780 (68%)	18,768 (93%)	6925 (97%)	

*IQR* interquartile range

*Tested using cuzick non-parametric test for trends

### Estimation of time-to-ART initiation over time

The median time from testing to initiating ART reduced from 17 days IQR (1–161) in 2004 to 2006 to one day IQR (1–14) in the period 2016–2017. Time to ART initiation increased with increasing age of the children with children aged from 5 to 9 years taking the longest time to be initiated on ART (14 days IQR (1–112) during the same period. Hospices had the longest time to ART initiation at 28 days IQR (1–357) while health posts had the lowest time to initiation at 1-day IQR (1–37) see Table 3.

**Table 3:** Incidence rate ratios time from testing to ART and incident rates of children living with HIV in Zambia from 2004 to 2017

Characteristic	Number initiating ART	Median time from Test to ART in days (IQR)	Rates of ART initiation 100 per month	Person time in years	Unadjusted test to ART IRR (CI)*	Adjusted from test to ART IRR (CI)*
Sex						
Male	33,211	10 (1–77)	14	18,909	1	1
Female	35,419	8 (1–77)	13.6	20,943	0.95 (0.93–0.96)	0.94 (0.93–0.95)
Years						
2004–2006	6217	17 (1–161)	7	9052	1	1
2007–2009	16,940	11 (1–111)	10	14,972	1.36 (1.32 –1.39)	1.34 (1.31–1.38)
2010–2012	19,780	10 (1–90)	15	10,823	1.98 (1.92 –2.02)	2.02(1.96 –2.07)
2013–2015	18,768	9 (1–50)	33	3777	4.55 (4.43–4.67)	4.62 (4.50 –4.74)
2016–2017	6925	1 (1–14)	106	421	14.5 (13.9–14.9)	15.2 (14.7–15.7)
Age group at test						
Birth to 17 months	12,141	1 (1–55)	16	6278	1	1
18 months to 4 years	18,953	8 (1–73)	14	10,860	0.91 (0.88 –0.92)	0.92 (0.90–0.94)
5–9 years	18,476	14 (1–112)	12	12,730	0.76 (0.74–0.77)	0.78 (0.76–0.80)
10–15 years	17,215	13 (1–77)	14	9898	0.86 (0.84–0.87)	0.86 (0.84 –0.88)
Health facility type						
Hospital	31,776	7 (1–87)	12	20,878	1	1
Health centre	36,177	13 (1–69)	16	18,352	1.26 (1–1.27)	0.99 (0.97–1.01)
Hospice	473	28 (1–357)	10	502	0.64 (0.58– 0.70)	0.42 (0.38–0.46)
Health post	133	1 (1–37)	14	118	1.14 (0.99 –1.31)	0.69 (0.60–0.80)
Province						
Central	5174	1 (1–42)	21	1977	1	1
Luapula	2677	1 (1–13)	24	880	1.22 (1.16 –1.27)	1.06 (1.01–1.11)
Copperbelt	15,423	1 (1–46)	14	8865	0.67 (0.65 –0.70)	0.59 (0.57–0.61)
Eastern	6758	14 (1–115)	11	4734	0.55 (0.53–0.57)	0.45 (0.44–0.47)
Lusaka	17,886	19 (1–84)	12	11,114	0.62 (0.60 –0.63)	0.62 (0.61–0.64)
Muchinga	1340	1 (1–18)	18	599	0.89 (0.84 –0.94)	0.80 (0.76–0.85)
North-western	2599	3 (1–58)	15	1354	0.73 (0.69 – 0.76)	0.61 (0.58–0.64)
Northern	2681	1 (1–17)	19	1129	0.93 (0.89 – 0.97)	0.69 (0.66–0.72)
Southern	9424	18 (1–136)	11	6684	0.57 (0.55 – 0.58)	0.54 (0.52–0.56)
Western	4668	16 (1–91)	14	2513	0.72 (0.69 – 0.74)	0.57 (0.54–0.59)

*IQR* Interquartile range, *CI* Confidence interval

*Tested using Poisson

**Fig. 2:**
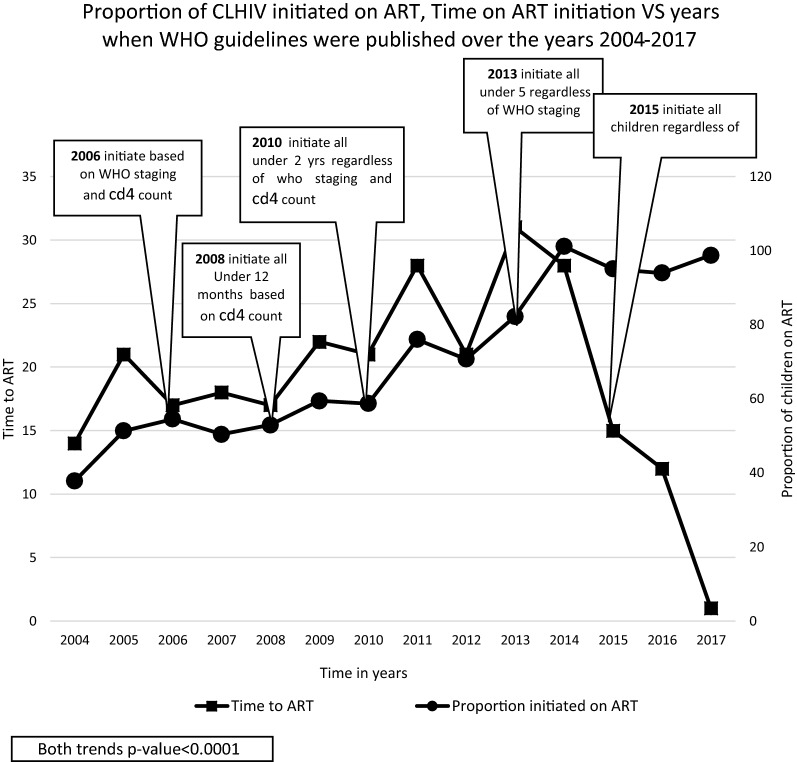
Graph showing how median time to ART and proportions of children initiated on ART have changed with changing WHO guidelines over the years (2004–2017)

Children were 15.2 times more likely to be initiated on ART in 2016–2017 compared to the period 2004–2006 (1RR = 15.2, 95% CI 14.7–15.7, p-value < 0.001) after adjusting for other variables (sex, province, age at HIV test, type of health care facility used). In estimating rates of ART initiation, the overall ART initiation rate was estimated at 0.14 per month or 1.68 per year, while the rate for ART initiation increased over the reporting period from 10 per 100-person months in 2004–2006 to 106 per 100-person months in 2016–2017. Since 2014 almost all children who tested positive for HIV have initiated ART, and the median time for ART initiation has dropped from 1 month in 2014 to 1 day in 2017 (Fig. 2). Furthermore, the ART initiation rates reduced with increasing age groups from 16 per 100-person months in the youngest age group, to 14 per 100-person months among children aged 10 to 15 years during the same period.

### Characteristics of children at ART initiation

Overall, the percentage of children who tested HIV positive who were initiated on ART increased over the years to 97% as of December 2017 from 52% in 2004–2006 (Table 2). Children from birth to 17 months recorded the lowest percentages of ART initiation among all the age groups with percentages under 20% over the period under review. In contrast, percentages of children initiating ART among the 10 to 15 age group were highest at above 25% with the highest percentage recorded between 2016 and 2017 (p-value < 0.001 z = 8.93). The proportion of children initiating ART in urban districts continued to be high over the period under review with initiations in urban districts maintaining more than 85% ART initiation rates, see Table 4.

**Table 4:** Baseline characteristics of children ART initiation in Zambia from 2004 to 2017

Characteristic	2004/2006 n = 6217	2007/2009 n = 16,940	2010/2012 n = 19,780	2013/2015 n = 18,768	2016/2017 n = 6925	P-value*
Age n (%)						< 0.001
Birth–17 months	467 (8)	2735 (16)	3 393 (17)	2 495 (14)	876 (13)	
18 months–4 years	1622 (26)	5235 (31)	5891 (30)	4846 (27)	1706 (26)	
5–9 years	2137 (35)	4625 (27)	5282 (27)	5379 (30)	1840 (28)	
10–15 years	1942 (31)	4230 (25)	4945 (25)	5468 (30)	2240 (34)	
Median age (IQR)	7 (4–10)	5 (2–10)	5 (2–10)	7 (2–11)	7 (3–12)	
Sex n (%)						< 0.001
Male	3085 (50)	8475 (50)	9569 (48)	8767 (47)	3273 (47)	
Female	3103 (50)	8446 (50)	10,192 (52)	9976 (53)	3641 (53)	
Place of testing n (%)						< 0.001
Hospital	3178 (51)	8983 (53)	9188 (47)	7774 (41)	2592 (37)	
Health centre	3002 (49)	7854 (46)	10,477 (53)	10,698 (57)	4105 (59)	
District type n (%)						
Rural	394 (6)	1981 (12)	2849 (14)	2807 (15)	854 (12)	< 0.001
Urban	5794 (94)	14,940 (88)	16,912 (86)	15,936 (85)	6060 (88)	

*IQR* interquartile range

*Tested using cuzick non-parametric test for trends

### Comparison of national estimates of children initiated into care with UNICEF and UNAIDS estimates

**Fig. 3:**
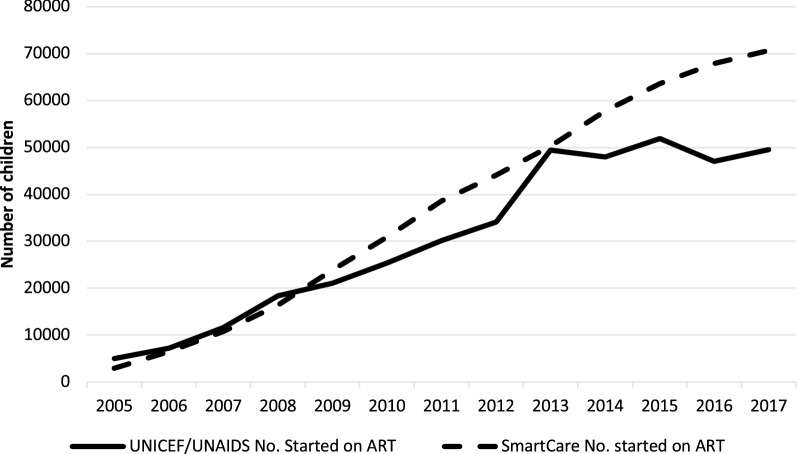
UNICEF and UNAIDS reported numbers vs SmartCare numbers of children on ART over the years

Cumulative numbers of all children initiating ART in SmartCare over the period under review and aggregate data reported in stocktaking reports published by UNICEF [22–26] and UNAIDS global reports and country data factsheets [27–31] show similar upward trends with minor differences among the data sources see Fig. 3. Our data shows slightly higher trends from 2009 to 2013 and from 2014 to 2017 despite the data coming from a percentage of health facilities in the country.

### Post regression estimates of time to ART initiation from multilevel regression clustering at facility level

The mixed random effects model showed significant variability in time to ART initiation by age at ART initiation and district type depending on the health facility used at ART initiation. Generally, time to ART initiation increased with an increase in age while it reduced by 25% (IRR = 0.117, 95% CI 0.099–0.138) in urban areas compared to rural areas see Table 5.

**Table 5:** Post regression estimates of mean time (in days) between testing HIV positive and ART initiation for children in Zambia between 2004 to 2017

Characteristic	Average days to ART initiation (CI)* crude estimates	P-value*	Average days to ART initiation (CI)** adjusted estimates	P-value **
Age				
Birth–17 months	58 (40–77)	Wald (3) = 2393 P < 0.0001	116 (84–148)	Wald (4) = 2412 p < 0.0001
18 months–4 years	134 (117–151)		191 (160–223)	
5–9 years	241 (224–258)		299 (267–330)	
10–15 years	262 (245–280)		320 (288–352)	
Sex				
Male	178 (160–196)	Wald (1) = 0.10 p > 0.05	111 (85–149)	Wald (5) = 2413 p < 0.0001
Female	179 (161–197)		108 (82–147)	
Place of testing				
Hospital	175 (141–209)	Wald (1) = 0.08 P > 0.05	110 (70–149)	Wald (5) = 2392 p < 0.0001
Health centre	181 (159–202)		120 (85–155)	
District type				
Rural	209 (175–242)	Wald (1) = 4.35 P < 0.05	116 (84–148)	Wald (4) = 2412 p < 0.0001
Urban	166 (145–187)		30 (17–57)	

Run using multi-level random effects regression clustering at facility level

*ICC* Intraclass correlation 11% (IRR = 0.117)

*Unadjusted model

**Adjusted for age and district of residence

## Discussion

This study describes the baseline characteristics of close to 100,000 children who were diagnosed with HIV over a 13-year period in facilities using SmartCare across Zambia. Our observations suggest that there was a substantial improvement in childhood HIV health care delivery and service uptake over years analysed. This is evidenced by improvements in specific parameters such time to ART initiation which reduced drastically during the same period. The data also shows the impact of the WHO guidelines over time with almost 100% of children diagnosed with HIV initiating ART since 2014. The 2013 and 2016 WHO guidelines which recommended immediate ART initiation for children have been instrumental in bringing down the delay in initiating ART, with most children now starting ART on the same day they receive their HIV diagnosis.

Despite these overall improvements there were lower rates of HIV testing and longer times to ART initiation for particular groups. Those aged 5–9 had the lowest rates of HIV testing and those aged 10–15 took longest to initiate ART treatment. Estimations of the numbers of children initiated on ART in our study were also found to be higher than estimates reported in UNAIDS and UNICEF documents. Our data shows that 97% of children diagnosed with HIV in 2016–2017 were initiated on ART which is higher than the UNAIDS estimation for Zambia that showed that 52% of children diagnosed with HIV were initiated on ART in 2017 [32]. The reasons for these differences were not the primary focus of this paper. However, disparity in percentages could be attributed to the differences in the reporting period and facility coverage. Our higher percentage is in line with reports from the Ministry of Health that showed a 65% increase in percentages of people initiating ART between 2016 and 2017 after the implementation of the test and treat strategy [33]. It is possible that our estimates have been affected by bias common when using routinely collected data such as missing and incomplete records, as shown in the drop in the number children that were diagnosed with HIV from a peak of 31,602 between 2007 and 2009 to only 7270 in 2016/17. This bias included loss in sample size leading to only complete case analysis and possibly under or over estimation of estimates [34]. We propose that this could have been due to effective PMTCT programs that led to 96% of pregnant women living with HIV receiving ART and a more than 50% reduction in the number of HIV positive children born from HIV positive women between 2011 and 2012 [35].

There were also challenges in ART initiation when disaggregated by age group with time to ART initiation increasing with increasing age. In addition, 11% variability in time taken to ART initiation in factors affecting children at ART initiation could be attributed to type of facility used at ART initiation. Our data showed that children aged 5 to 9 years had the longest time to ART initiation and were less likely to be initiated on ART compared to children from birth to 17 months. This could be because until 2016, children above 5 years could only be initiated on ART once their CD4 count was below 350 mmol/l thereby increasing time to ART initiation. Another reason for the delay could be some AIDS defining conditions like TB that have overlapping symptoms and require initiation of ART after treatment of the condition is tolerated [36–39]. In addition, children above 5 have less contact with the health facilities after completion of the Mother and child health (MCH) clinics immunization and growth monitoring schedule. Therefore, diagnosis for this age group is more likely after presenting with acute illness at health facilities [39]. Another area with challenges is the testing related dynamics where children aged 10 to 15 years had the lowest rates of HIV testing. This could be because until recently long-term survival of children not on ART following MTCT were thought to be uncommon and so testing strategies for this age group were not emphasized or were less robust [10, 39]. Most children in our study were tested in hospitals in 2004–2006. However, this trend reduced between 2015 and 2017 with increasing testing in the other facilities types such as hospice and health posts showing the need for increased EID services at all health facility types [40]. We also found that more female children were tested over the study period. Reasons or this are beyond the scope of this study. However, sex related differentials among children testing for HIV have been reported in earlier studies in Zambia which showed more males testing for HIV among hospitalised children [10].

### Strengths and limitations

To our knowledge, this paper is the first to show long term changes in the characteristics of HIV infected children from facilities across Zambia using routinely collected SmartCare data. The main strength of this study is the large sample size and the long follow up period which makes our findings generalizable to the whole population. In addition, we analysed data from public, private, military and faith-based health facilities that utilize SmartCare at different levels of the health care delivery system. Our analysis was merely descriptive which made it difficult to attribute causality and a more robust analysis is required to understand the trends in diagnosis and initiation onto ART.

The incidence of children initiating ART decreased with increasing age, showing the need to promote community-based programs for routine HIV testing in children above 5 years. Facility-based same-day test and treat should also be supported to reduce the drop out of children between HIV diagnosis and ART initiation. The magnitude of missing data may have been small for an epidemiological analysis but is alarming if proper patient follow-up and care is to be achieved. The Ministry of Health needs to ensure data capturing monitoring and reporting are easier for health facilities to complete in order to improve levels of data completeness, accuracy and quality.

## Conclusion

WHO has spearheaded the fight against HIV and AIDS providing guidelines for both testing and treatment of CLHIV. Zambia has seen substantial increase in proportions of ART initiation among CLHIV and reduction in time to ART initiation which suggests successful implementation of WHO adapted national testing and treatment guidelines and improvements in HIV programming. These efforts and should be supported as they have saved the lives and improved the quality of life of CLHIV in Zambia. However, age related problems with testing suggest that there are critical areas that must be targeted for improvements. These problems might be showing a gap in health system functioning associated with access. There is therefore need to address this and target hard to reach groups if improvements are to be sustained.

## Data Availability

The datasets analysed during the current study are not publicly available due the data were collected as routine patient data by the Zambian Ministry of Health who are the owner of the data therefore dataset cannot be shared online. Any further information on the use of dataset should be addressed to the Zambian Ministry of Health through the office of the Permanent Secretary at info@moh.gov.zm. However, for replication and verification of the study, guidance on the dataset and variable names mentioned in the study’s methodology section of the manuscript is available from the corresponding author on reasonable request.

## Abbreviations

AIDS: Acquired immunodeficiency syndrome
ANC: Antenatal clinic
ART: Antiretroviral therapy
EFV: Efaviranz
HB: Haemoglobin
HIV: Human immunodeficiency virus
IQR: Interquartile range
LTFU: Lost to follow-up
MTCT: Mother to child transmission
NVP: Nevirapine
PIs: Protease inhibitors
PMTCT: Prevention of mother-to-child transmission
UNAIDS: United Nations programme on HIV/AIDS
UNICEF: United Nations children emergency fund
WHO: World Health Organization
